# Speak for yourself: usability and acceptability of audio diaries to explore physical activity, sedentary and sleep behaviours of those living with severe mental illness

**DOI:** 10.1186/s44167-024-00058-4

**Published:** 2024-09-11

**Authors:** Ilaria Pina, Philip Hodgson, Kirstie Anderson, Emily J. Oliver

**Affiliations:** 1 Newcastle University, Newcastle upon Tyne, UK; 2 Tees, Esk and Wear Valleys NHS Foundation Trust, Darlington, UK; 3 York St John University, York, UK; 4 Newcastle upon Tyne Hospitals NHS Foundation Trust, Newcastle upon Tyne, UK

**Keywords:** Mental health, Co-production, Physical health, Qualitative data, Inclusive, Participatory approach

## Abstract

**Background:**

People living with severe mental illness (SMI) face significant health inequalities, including reduced quality of life and life expectancy. Evidence has shown that people living with SMI are highly sedentary, face challenges when seeking to engage in physical activity (PA), and experience sleep difficulties. Motivation, mood and energy have been identified as critical determinants of these behaviours. PA and sleep are traditionally measured in isolation using quantitative approaches, limiting our understanding of the contexts and interactive ways in which these occur, especially for this population. Here, we adopted a flexible and holistic approach, using audio diaries to explore the usability and acceptability of capturing movement behaviours in people living with SMI.

**Methods:**

This study employed a qualitative design. Data were collected with 10 participants self-identifying as living with SMI, who completed 7-days of audio diaries, pre and post diary use interviews. Reflexive thematic analysis was used to analyse participants’ movement behaviours and their experiences of using the audio diaries.

**Results:**

Audio diaries were perceived as acceptable to participants and their use for data capture was feasible, with participants experiencing their use as a flexible and empowering method of data capture. Within the exploratory data generated we identified four themes relating to participants’ movement behaviours: finding themselves in a “vicious circle” with physical and mental issues impacting movement behaviours; a daily internal fight and dialogue concerning fear of feeling guilty and wasting time; a determination to “not let fatigue win” by pushing through the day; and the mixed effects of understanding the importance of movement behaviours yet finding it challenging to engage.

**Conclusion:**

Audio diaries offered an easy to use and relatively inclusive means of exploring movement behaviours for people living with SMI, especially their context and interrelated nature. Our findings reinforced the well-established link between mental and physical health, and their influence on 24 h movement behaviours, identifying population-specific challenges derived from medication side effects, rigid engagement opportunities, and illness symptoms. Given this, co-production involving individuals with lived experience is crucial for developing tailored recommendations and support to promote sleep and movement among those living with SMI. We emphasized the need for holistic measurement approaches and opportunities that consider the interconnected impact of disrupted sleep and movement.

**Supplementary Information:**

The online version contains supplementary material available at 10.1186/s44167-024-00058-4.

## Background

Severe mental illness (SMI) is a major cause of years lived with global disability [1], with often detrimental and substantial impacts on daily life functioning and quality of life. In addition, people living with SMI are over three times more likely to have a physical health condition, and their life expectancy is 15–20 years lower than the general population [2]. The development of comorbid physical health conditions in people living with SMI creates an important healthcare challenge for the UK’s National Health Service [3], as it aiming to expand access to physical health assessments and complimentary evidence-based intervention. This disparity in health outcomes is partly due to modifiable health behaviours, such as high levels of physical inactivity and sedentary time [4, 5], which in turn lead to disrupted sleep quality and quantity [6]. Here, we considered a broad definition of SMI aligned with the one adopted by the UK Office for Health Improvement and Disparities [7], allowing people to self-identifying as having a mental health condition severely impacting their life and activities [8]. This is aligned with previous approaches that consider mental health as a societal issue, with relationships to social determinants of health, and arguments that the severity of impact of mental health conditions on the individual is often driven more by circumstance than diagnosis [8].

Physical activity (PA) is a modifiable risk factor and interventions are a relatively low risk and accessible way of reducing physical health problems in people living with SMI. However, being physically active has been shown to be a complex behaviour both in general and especially for people living with SMI [4]. Medication side-effects such as sedation and fatigue, low or suboptimal motivation, and co-existing physical health conditions, as well as illness-related symptoms, are physical and psychological barriers to PA engagement. These are also potential reasons for high levels of sedentary time [9, 10]. In addition, sleep disorders are common in individuals living with SMI, with up to 90% of adults reporting abnormal sleep behaviour, such as increased sleep onset latency, insomnia or hypersomnia [11]. Sleep can also be disrupted due to the use of psychotropic medications impacting sleep duration and architecture [12], alongside weight gain as a direct side effect that increases the risk of obstructive sleep apnoea [13]. Given the above, we argue that PA, sedentary time, and sleep are helpfully considered as interactive components impacting on quality of life and wider health outcomes.

Despite the presence of several guidelines and toolkit on physical health and PA in the UK [14, 15], these tend to focus on single behaviours or pay relatively minimal attention to the interplay between movement behaviours. However, prior research has shown a preference for holistic approaches that focus on balance across behaviours and life contexts [16]. Researchers support the idea of moving beyond the target of more PA towards finding a healthy balance between movement behaviours [17]. Dedicating time to PA requires a compromise, as time for PA must come from other activities within the day, likely from sedentary behaviour or sleep [17]. Indeed, to understand PA holistically, it is important to explore the decisions on time allocation alongside the type and context of activities [17]. Existing measurement tools and approaches, often validated with a general population sample, offer little insight in this regard, especially for populations who face multiple context-specific barriers to movement. There are still few studies that have adopted 24 h approaches, limited qualitative approaches applied overall (narrowing which contextual variables are captured or viewed as important), and no work to date that focuses on 24 h movement behaviours for people living with SMI.

The challenge of implementing health behaviour approaches in those with SMI mean that a more nuanced understanding of “active living” as healthy balance of movement behaviours incorporating both sleep and rest time is needed. To this end, here we adopt a participatory approach [18] with the use of audio diaries to capture the type and context (opportunities where people are moving but also the context of their wider life and condition status) of all three interrelated movement behaviours in this population for the first time. Through co-developing a participatory approach, we will gain a clearer understanding of how people living with SMI allocate their time to movement behaviours and what it is important to consider in this population. Thus, helping policymakers to tailor strategies and outcomes to this population with more feasible intervention goals such as move towards a healthy daily balance by reducing sedentary time or by supporting sleep improvements [17].

Audio diaries provide the opportunity to capture phenomena and perspectives otherwise inaccessible to researchers because of time, context, and convenience [19]. This approach allows the participant to interpret the research question in the context of their own daily life with a sense of comfort and safety [19]. The fluidity in speech during data collection enables an immediate response to questions [20, 21], and has the potential to allow the researcher to uncover details about participants’ experiences in a safe and engaging way that is particularly important when working with groups that have historically experienced stigma and discrimination [22].

Assessing movement behaviours is a challenge in any population and requires varying and flexible approaches. People living with SMI are usually underrepresented in research samples [22], generally it is unclear if and how the interconnection of movement behaviours apply to them and how this could be appropriately assessed to capture their perspectives and life contexts. As adults living with SMI are valuable experts of their own behaviour and daily life [23], their perceptions are essential when measuring their movement behaviours. Previous studies have assessed the feasibility and validity of accelerometers [24], self-completion diaries and questionnaires [25] to measure behaviours in people living with SMI. Quantitative measures are able to assess different dimensions of PA, along with a variety of metrics such as number of steps, minutes of activity, intensity of activity, and bouts of activity [26]. However, the measurement of PA in people living with SMI presents unique challenges given heterogeneity and differing experiences such as condition-related fatigue, concentration or motivation challenges [4], as well as additional barriers that are overrepresented in this group relative to the general population, for example digital literacy barriers [27], general literacy levels [28], and other physical health barriers [10]. Some of these challenges are also present in people with other conditions including cancer or diabetes [29], highlighting potential wider applications of this approach for other groups and the general population. It is therefore important to consider whether this measurement approach could be appropriate and engaging: is it acceptable to participants, especially those facing the challenges of managing a chronic condition, and is it feasible for use by those living with SMI? As such, here we piloted the use of interviews and audio diaries to answer our primary research question on the usability and acceptability of audio diaries in people living with SMI as data collection tool from a participant perspective. In doing so, we generated exploratory data to examine which aspects of movement behaviours and their contexts are important to consider in this group. to provide insight to inform future data collections in this area.

## Methods

### Public involvement and recruitment

Six members of the public with lived experience of SMI were involved prior to the start of the study to design and inform the inclusion criteria, recruitment strategies, interview schedules, and the audio diaries. They were recruited via voluntary organizations, mental health charities, community services and were reimbursed for their time based on the National Institute for Health Research guidelines [30].

Ten participants who had not contributed to the public involvement stage, living in the community, aged 18 years or over, and living in and/or accessing services in the North of England (UK) took part in the study. Aligned with calls for a broader definition of SMI [8], participants self-identifying as having a severe experience of mental illness [8] were invited to take part in the study. Participants were recruited via invitation emails to charities supporting mental health, third sector and community organisations for individuals living with SMI. Exclusion criteria were those lacking the capacity to consent and non-English speakers. Demographic data were not collected, instead the researchers asked participants to provide a self-written free-text qualitative description, allowing them to share with the research team personal details that they thought were important in relation to the research question. Researchers provided an example of qualitative description in the follow up email after the entrance interview took place.

Ethical approval was granted by the Population Health Sciences Institute at Newcastle University (ref. 2591/34063). Participants were provided with contact details of the researchers and mental health charities in the study material in the event they felt they required additional support.

### Data generation

#### Interviews

Participants completed two in-person or online (completed via Microsoft Teams) semi-structured interviews before and after the 7-day period of audio diaries. Participants with lack of access to a computer or lacking digital skills were firstly contacted via phone call and/or received instructions on using the online platform via post, a participant was also supported by the partner during the process. Alternatively, participants were offered in-person interviews. The entrance interview sought to explore participants’ past experiences and future expectations around healthcare and community support for PA, sedentary time, and sleep. The exit interview inquired about participants’ experiences on the use of audio diaries, allowing the researchers to gain feedback about this approach in the context (here defined as opportunities where people are moving but also the context of their wider life and condition status) of movement behaviours, for improving the method, its implementation and application for future studies [20]. Questions for both the entrance and exit interviews are presented in the Additional file 3.

#### Audio diaries

After the entrance interview, participants were provided with a voice recorder (VN-541PC, Olympus Imaging Corp, Taiwan) and with equipment instructions to collect data for a 7-day period. The specific model of voice recorder was chosen due to its “instant dictation” option allowing participants to instantly start recording even when the power was off. In this study, audio diaries were used to describe movement behaviours. The researchers performed a facilitative role to allow participants to use the audio diaries in a way that suited them throughout the research. Indeed, participants chose when to complete the audio diary either throughout the day or at the beginning/end of each day, with no minimum data entry required. Participants could make multiple recordings throughout the day. They were reimbursed for their time with an incremental reimbursement based on the number of days of data entry provided [31].

Based on the six public engagement sessions, adjustments to the study design were made: participants were able to express a preference to receive a reminder to complete the audio diary. If yes, they could choose how often they wanted to receive the reminder and whether via text or email. The research team provided an audio diary guide to the participants (Additional file 1). In this study document, a brief summary of the study, definitions of PA, sedentary time and sleep, and a list of prompts in relation to the research question were included. As suggested by previous research, participants were asked to dedicate as much or as little time to the prompts as they deemed relevant [20] to allow for a balance between a structured approach and autonomy in recording diary accounts.

### Theoretical assumption and methodological approach

This study was grounded ontologically in relativism and epistemologically in constructionism [32], with an experiential orientation to data interpretation to capture and explore participants’ perspectives and understandings on 24 h movement behaviours [32]. A predominantly inductive orientation to data, with both semantic and latent coding, was adopted here with the analysis driven by data content, limited only by a focus on constructing themes of relevance to the research question [33]. The research team involved two physiotherapists from different backgrounds (general medicine, mental health, and neuro rehabilitation), a sleep specialist, and a psychologist with expertise in behavioural science and mental health.

### Data analysis

All interviews, audio recordings and audio diaries were transcribed verbatim by a member of the research team (IP) and reviewed by the research team to ensure clarity and accuracy of transcription. Participants were assigned a unique code number and all other identifying information was redacted to protect privacy and anonymity. Data were analysed with reflexive thematic analysis due to its suitability and flexibility to analyse data derived from a pluralistic approach to data generation [34], but also to allow both descriptive and interpretative accounts of data, allowing for both inductive and deductive theme generation. The lead author familiarised with transcripts to note initial features of the data and potential points of analytic interest. The initial coding produced several hundred codes, that were then clustered into five groups: sleep, PA, feelings, relaxing activities, and reflections on the use of audio diaries. We then moved to the interpretation of aggregated meaning across the dataset [32] to combine codes according to shared meanings into candidate themes. These were further refined and named through discussion across the research team.

## Results

### Participants

Ten participants completed the study process (entrance interview, 7 days of audio diaries, and exit interview). However, we excluded one participant from data analysis as they did not record audio diaries but paper diaries during the week of data generation. A total of three participants preferred to be interviewed in-person, while the remaining 6 preferred to be interviewed online. Participants’ self-written free-text qualitative anonymised descriptions are provided in Additional File 2. Here, we provide a brief composite summary: some participants had lived experience of mental health conditions or within their families (including depression, anxiety, post-traumatic stress disorder, attention deficit hyperactivity disorder, psychosis, and personality disorders). Some have also experienced trauma or violence in their past, contributing to their mental health challenges. Professionally, some participants have worked or are currently working in mental health-related fields, such as nursing, peer support, or social care. Family dynamics vary among the participants, with some being single parents, widowed, or married with children. Several have adult children, while others are caregivers to their partners or have experienced significant loss.

### Audio diaries: quantitative usage data

Quantitative results summarising the usage of the audio diaries are presented in Table 1. Of the 9 included participants, 8 recorded 7 days of audio diaries while one participant recorded 5 days of audio diaries due to issues in operating the recorder. A total of 110 recordings were received overall with a total of 3 h and 55 min of recording across participants. The minimum number of recordings per participant was 5 and the maximum was 22, with 4 participants providing multiple recordings per day of data collection.

The average recording duration was 2.5 min, with the shortest duration average being 20 s and the longest duration average being 5 min. The most common times to record audio diaries were morning (between 6:00 and 12:00) and late evening (between 20:00 and 00:00) with 38% and 36% of total recordings completed during these times respectively. The least common times to complete recordings were afternoon (12:00–16:00) and overnight (00:00–06:00).

**Table 1:** Quantitative usage data from audio diaries for each participant

	Characteristics of data entries					Number of recordings completed within each timeslot					
Participant ID	Reminder frequency	Days recorded (*n*)	Total number of recordings (*n*)	Total recording duration (mm: ss)	Average recording duration (mm: ss)	Morning (*n*)	Afternoon (*n*)	Early Evening (*n*)	Late Evening (*n*)	Overnight (*n*)	
1	1 per day	5	5	19:11	03:50	0	0	4	1	0	
2	1 per day	7	7	37:05	05:18	0	0	2	5	0	
3	1 per day	7	8	16:03	02:00	0	0	2	6	0	
4	2 per day	7	16	10:50	00:41	8	2	5	1	0	
5	1 per day	7	22	56:22	02:34	15	1	2	4	0	
6	no	7	8	24:46	03:06	6	0	0	2	0	
7	no	7	7	34:29	04:56	0	0	1	6	0	
8	1 per day	7	18	36:05	02:00	4	3	1	8	2	
9	no	7	19	20:00	01:03	9	0	3	7	0	

Time of the day defined as: morning (06:00–12:00), afternoon (12:00–16:00), early evening (16:00–20:00), late evening (20:00–00:00), and overnight (00:00–06:00)

### Audio diaries: qualitative usage data and acceptability

Here, we focused on participants’ views and experiences of the audio diaries method from the audio diaries themselves and their post-use interviews. In sum, the data below demonstrate that participants found the audio diaries to be an acceptable research tool, moving quickly from initial nervousness to empowered engagement. Further, the diaries were tools for reflection and in themselves were perceived to offer benefits. We identified some evidence that audio diaries would offer time and accessibility improvements relative to paper-based tools.

#### Characteristic 1. Audio diaries allow for a dedicated time for reflection – potential for being a therapeutic tool

Participants described the audio diary process as helpful in reflecting upon their movement and sleep routines. Indeed, it was seen as providing them with a reflective outlet for the challenges of committing to take part in structured PA, maintaining appropriate sleep hygiene and enabled them to have a follow on their issues or seek for help: “*You can also reflect on: so what? What can I do? Because it’s been recorded it*,* it’s almost like it clears the mind because it makes key things stick in my mind and makes it easier for me to be able to sort them out*,* get them in perspective*,* but just because I’m not talking about the mental issues themselves*,* I’m talking about how do how do I continue to structure in that physical activity when things get really*,* really bad and how well it works when things are manageable or fairly manageable. It’s a really useful exercise.”* (Participant 5).

Some participants felt this was therapeutic, as the audio diary offered a cathartic opportunity to vocalise what they needed to say: “*I then found it absolutely amazing*,* really sort of continued to do it because it gives you a reflection of everything you’ve done in that day. You’ve got it off your chest and said it instead of it playing over your mind.”* (Participant 2). This approach provided an opportunity for participants to share their reflections and thoughts without any expectations of providing a certain amount of information as per “filling a box” on paper. Also, the lack of consistent “visual feedback” as from paper diaries, avoided further self-scrutiny or review: “*It’s not triggering me because I don’t have to….like*,* I’m not listening to it. If I write it all down*,* I’d say have that piece of paper every single day*,* seven days. So then I’d be like*,* oh*,* yeah*,* that happened. I mean*,* is this consistent visual feedback from the paper that you get like maybe did not write enough in the box or there is there was not enough space and you keep reminding from the paper.*” (Participant 2).

#### Characteristic 2. The method allowed to overcome time and accessibility constraints

Participants shared the positive aspect of the practicality of the audio diary method. Completing the diaries was experienced as convenient, easy and instantaneous: “*You can do it whilst you’re doing something else. Like whereas if you have to take the time to sit down and write they*,* I mean I don’t mind writing*,* it’s fine. Well*,* for me*,* like we all quite have busy lives. You know what I mean? And we all have other commitments and stuff. So for me this it’s much more convenient to be able to just speak into a recorder and document it that way.*” (Participant 6). Also, audio diaries allowed participants to share more details and information compared to paper diaries: “*I think the good thing with the recorder is like in a 10 minute recording you can fit so much and like even though some days I was only recording for 10 minutes like was able as I was able to say a lot*,* wherever in a day 10 minutes of writing is nothing*.” (Participant 1). The approach also allowed the research to be inclusive by overcoming visual impairments and physical issues (e.g. shoulder injury): “*It made me feel empowered and full of energy. Ohh well*,* I’m not doing anything for a long time*,* for my eyesight*,* nothing worse. It’s becoming quite difficult*.” (Participant 8).

#### Characteristic 3. Challenges of audio diary use

Negative responses and experiences from participants about the audio diaries were relatively few. The most reported concern related to participants nervousness in providing “right” data and that their accounts were going to look repetitive: “*I think it was more nervous of*,* like*,* right. What*,* what if I like*,* say*,* the right thing or what if I say the wrong thing or like?*” (Participant 2) and “*And I think I did feel a bit like*,* I’m just gonna update or not much because I haven’t done too much so there’s not loads I had to discuss*.” (Participant 10). Some participants described also how the awareness achieved through the recordings about movement and physical activity occasionally has been perceived as discouraging. However, in some this prompted the opportunity to consider change: “*It’s like you*,* you start to feel low about how much you’re actually not moving*,* but then you take the positive out of it and you start to think like what*,* what do you need to change and improve and things like that?”* (Participant 9) and “*I think from doing it is made me think a little bit*,* uh*,* about how much it exercise or like stuff that I actually do and when I was recording it*,* I was thinking actually I didn’t do very much. So it highlighted to me really that I should probably do more exercise*” (Participant 10).

### Understanding interactions between sleep, PA, and sedentary time

**Fig. 1:**
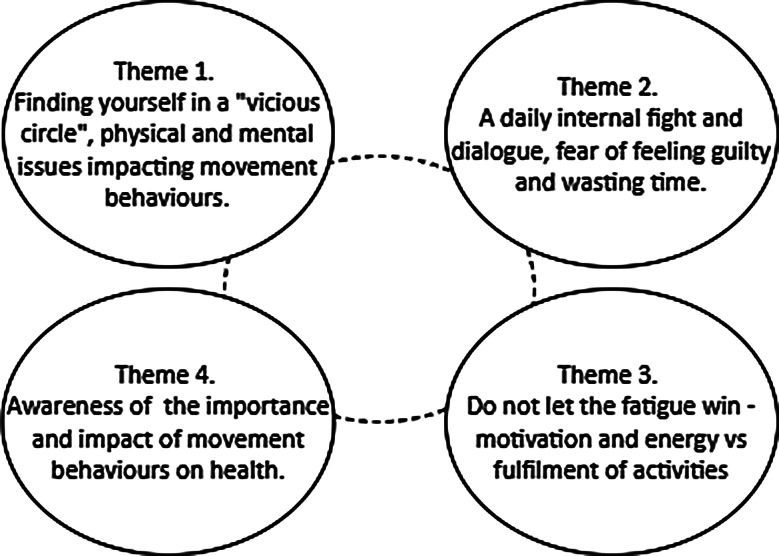
Map of themes generated through reflexive thematic analysis related to our understanding of 24 h movement behaviours in people living with SMI

Here, we address the contributions of the entrance interviews and audio diaries to the secondary research question exploring PA, sleep, and sedentary time in people living with SMI. Four overarching themes were generated from the data, a summary is illustrated in Fig. 1.

#### Theme 1. Finding yourself in a “vicious circle”, physical and mental issues impacting movement behaviours

In this theme, we noted participants’ experiences of a “vicious circle” emphasising the intersection between physical and mental aspects of health in daily life and further supporting the idea that physical health problems increase the risk of poor mental health, and vice versa. Participants described how the interaction between physical and mental health impacts health-related behaviours, including sleep and PA: “*Unfortunately*,* it’s a vicious circle for me. I’ll get physically unwell. Then I’ll get mentally unwell and then I’ll not sleep and then I’ll not eat*,* and then I’ll not exercise and then I’ll not do anything and it just goes round and round and round until I’m well enough physically to pull myself out mentally*,* and then it starts coming back to us*.” (Participant 1 on movement and sleep). Participants also described the presence of physical side-effects of medication and/or physical conditions limiting their motivation and ability to move. “*I think for me*,* one of the side effects of the medication I take as well makes you really drowsy. So*,* I’m usually quite tired. A lot of the time*,* and so that doesn’t help me even really cause that sort of adds to my lack of motivation.*” (Participant 9 on movement).

Alongside physical symptoms, events perceived as stressful (such as memories of trauma or unexpected medical visits) and the anxiety deriving from them, led to negative experiences of overthinking/active mind further impacting willingness to move: “*I’ve got still all playing in my mind from the hospital*,* so I’ve had no motivation at all today to do anything.*” (Participant 2 on movement). This last emotion-based aspect also impacted sleep with participants’ experiencing many nocturnal awakenings and nightmares, contributing to feelings of tiredness and fatigue. This lack of good quality sleep led to worsening in mood, further impacting ability and willingness to move.

#### Theme 2. A daily internal fight and dialogue: fear of feeling guilty and of wasting time

This theme brought to light participants’ experiences of an internal dialogue on the choice of staying in bed or starting the day. Most of the participants shared difficulties in nocturnal sleep and the challenge to get out of bed in the morning to face the day: “*I was debating whether to stay awake or go back to sleep or…Thinking well*,* I’ll be knackered all day*,* but I just thought no*,* just stay awake and just push through the day*.” (Participant 2 on sleep). Mood and stress in dealing with daily activities also contributed to a challenging start of the day: “*If I don’t force myself to do something*,* I’d just sleep and it’s not good. So*,* I’m gonna get myself sorted now*,* get myself up out at the house*,* and once I’m out*,* I’ll be fine. I know I’ll be fine. It’s just that initial pushing yourself to get ready and get out when all you want to do is go back to bed and go to sleep.”* (Participant 6 on all behaviours). Some participants shared how this internal fight requires to force themselves to be active to avoid feelings of guilt and the fear of “wasted day”: “*Because if I don’t force myself to be out and be active*,* I will just literally go back to bed and spend the whole day in bed and then I’ll get angry that I’ve wasted the day*.” (Participant 8 on all behaviours) and “*So*,* I had parents even last night and then I could have got a lift back*,* but I said no*,* and I chose to walk. I just needed to get out and get some fresh air*,* it’s amazing how guilty I can feel as well for not being very active.*” (Participant 6 on movement). Also, motivation played a decisive role in this internal dialogue: “*I know from previous times if I do exercise afterwards*,* I feel quite good doing it and pleased that I did it. So*,* it’s just like to get you the motivation to do it in the first place*.” (Participant 10 on movement) and “*I have no motivation to do anything*,* just wanted to stay in bed all day. By myself. Went to the to the table was going to do jobs for my mum today but then didn’t end up because just couldn’t be bothered to do anything*.” (Participant 2 on sedentary).

#### Theme 3. Do not let fatigue win – motivation and energy vs. fulfilment of activities

This theme enables an understanding of the fulfilment of activities as a driver of positional change in terms of their wellbeing status, with significant impact on perceived mental health and emotional wellbeing: “*I’m just trying to push through it (*the day*) because if I let the fatigue win then I will just end up doing nothing and I will end up in bed all day*,* every day*.” (Participant 6 on all behaviours). Being active was rewarded with positive feelings and sense of achievement afterwards. In particular, activities related to a feeling of commitment and/or with a purpose and/or tangible outcomes such as walking with the dog, household chores, DIY, and shopping led to positive experiences: “*I’ve done a long walk today. I couldn’t really be bothered*,* but my dog needed to go out and we had the most pleasant walk. All the leaves down and very pleasant. So yeah. So*,* and I feel it lifted my mood.”* (Participant 4 on movement) and “*I thought if I just don’t keep myself going. Then I’ll just end up just doing nothing and then it just mentally makes me worse. So*,* I just thought*,* you know what? Do some decorate and clean the flat. Makes me happy.”* (Participant 2 on movement and sedentary).

The enjoyment from being active was perceived to positively contribute to mental wellbeing: “*I’m really glad that I did my walk. It does. It really does help. The enjoyment from walking just helps to ground me and clear my mind*,* how much of that is psychosomatic? I don’t know*,* but it doesn’t matter as long as it helps.*” (Participant 5 on movement). Some of the participants also shared how the benefits from participating in activities outdoors or together with others had a positive impact on their emotions and their mental health: “*I’ve been for a long walk down at the coast with friends. Oh*,* looks lovely down there and it really helped. And I was just saying to my friends*,* what a difference*,* what I’m like today compared to what I was like when I was down*,* I was in a really bad way on Friday.*” (Participant 4 on movement) and “*I know personally that when I don’t get outside and I don’t do like*,* I don’t get fresh air and I don’t actually get my steps in and stuff*,* it affects me and it affects my mental health.”* (Participant 6 on movement).

#### Theme 4. Awareness of the importance and impact of 24 h movement behaviours on health

This theme highlighted participants’ awareness of the importance of having good quality sleep and being physically active. Participants placed a high value on getting good quality sleep with an emerging consensus that poor sleep significantly undermined their mental health and ability to function during the day: “*I’ve been struggling with sleep for a couple of days now. And it is majorly affecting my sleep routine which then in turn is also affecting like obviously my daily activities. The night before last I was up about four or five times throughout the night. And yesterday morning I was completely exhausted.”* (Participants 6 on all behaviours). The lack of good quality sleep led to avoidance behaviour towards daytime napping defined as a “fighting chance” to sleep more and better during the night: “*I’m not good at napping through the day because then I find that if you nap during the day*,* you don’t sleep on the nighttime. I don’t sleep on the night-time anyway*,* but that’s at least well*,* I would give myself a good fighting chance of sleeping by not sleeping through the day*.” (Participant 2 on sleep). Alongside the pressure to get appropriate sleep, participants’ experiences focused more on desirability and hope for good sleep, rather than tolerability of poor sleep: “*I’ll sleep*,* I do hope every night. Doesn’t seem to work right but that’s all well and good in the hoping*.” (Participant 2 on sleep).

Participants showed awareness and knowledge of the public health messaging in relation to PA targets and recommended amounts: “*I was active for 70 minutes according to my watch. So*,* I was active for more than the recommended 30 minutes a day*.” (Participant 6 on movement). Movement was seen to provide holistic health benefits, with walking (particularly outdoor) being a commonly reported PA being described as an easy and affordable option to move: “*Walking is what I’ve always done. And I know that it’s good exercise as well*,* but it’s good for the soul mainly.”* (Participant 5 on movement) and “*I decided to do physical activity today because I could have got a bus or a taxi. I could have got a taxi to the hospital. But I’ve decided to walk instead to the bus at least. I just decided to do this because it would be easier and more affordable than getting a taxi.”* (Participant 3 on movement). However, participants also described the difficulties in engaging in movement commitment regardless of the physical and mental benefit derived from it, with an evident need of being flexible to avoid additional pressure: “*And I think it goes to show how you can’t stick to a really rigid programme. Either you can do your best*,* but it’s…I think when things are not good and when we’re not well in our minds*,* it’s hard to move. It doesn’t need to be an added pressure that we put on ourselves as long as we have something structured in and something that we know that we should be doing that will help us. That’s the most important thing.*” (Participant 5 on movement).

## Discussion

This study explored the acceptability and usability of audio diaries as data collection tool concerning how movement, sedentary time, and sleep were experienced in a 24 h context for people living with SMI. To our knowledge, this is the first study to use audio diaries to collect data on movement behaviours in a 24 h framework in any population. Audio diaries were found to facilitate reflection among participants on their movement and sleep routines. Despite some initial anxiety, participants highlighted the practicality and accessibility of this method, as well as that it provided a positive and potentially beneficial research experience. We identified participants’ experiences of a “vicious circle” describing the reciprocal and challenging influence of physical and mental health status (including impacts on motivation, energy) on their movement behaviours. We found that engaging in purposeful movement activities had a positive and transformative influence on health, providing participants with a sense of fulfilment and benefits on emotional wellbeing. Across our sample morning waking was a particularly critical context, with an internal struggle experienced between staying in bed and the need to force themselves to move and face the day to avoid negative impacts of being sedentary on mental wellbeing. Participants were clearly aware of the interactive role of movement and sleep behaviours but despite acknowledging the benefits, participants described challenges and frustrations when seeking to adhere to structured activity regimens or sleep hygiene recommendations, underscoring the importance of flexibility and self-compassion in pursuing holistic wellbeing.

When participants described their daily experiences in relation to movement and PA, the impact of motivation and tiredness became evident. In line with previous evidence [35], lack of motivation, or suboptimal motivation, was presented as a barrier to PA engagement and wellbeing. Motivation for PA among the group often appeared to be driven by guilt rather than enjoyment, raising also concerns about the sustainability of these activities and impact on wellbeing. Drawing upon self-determination theory, the role of guilt in motivating behaviour aligns with the concept of introjected motivation [36], with the imposition of pressures by feelings of guilt or self-criticism while PA outcomes are personally important (e.g. beneficial for physical health and mental wellbeing) [37]. Our findings support the idea that to possibly increase PA engagement in SMI there should be a shift of policy promotion and public health messaging towards emphasizing enjoyment rather than targets for time and/or intensity. Participants shared their thoughts on the importance of engaging in PA, however medication side effects and dealing with illness symptoms (both physical and mental) often hindered their ability and willingness to move. Therefore, here we suggest a need for flexible engagement opportunities, similarly to previous research, including activities such as walking [38], being outdoor [35, 39], and small group activities [35], along with the necessity for specific guidelines tailored to this group. Moreover, there is a call for greater involvement and co-production of recommendations involving individuals with lived experience of SMI to ensure their needs and perspectives are adequately addressed. Some good practice examples of meaningful co-production of PA recommendations are emerging [40, 41].

Participants, echoing findings from previous research [42], placed a high value on getting good quality sleep. They also described sleep issues with frequent awakenings, nightmares, restless nighttime sleep and how the accumulated sleep loss led to an amplification of negative emotions, diminished energy impacting their daily life and overall health. Occurrence of comorbid sleep and circadian rhythm disorders are commonly experienced by individuals living with SMI [43], affecting emotional and cognitive functioning [44, 45]. Nonetheless, the reluctance among participants to incorporate planned voluntary daytime naps, despite evidence suggesting benefits on cardiovascular outcomes and cognition [46, 47], highlights the need for interventions and public messaging that integrate restorative activities into a new nuanced and broader concept of active living. There is a clear imperative for a holistic approach to sleep health promotion which also addresses the interconnectedness of physical and mental wellbeing.

Participants emphasised the opportunity provided by audio diaries for a dedicated time for reflection, recognising a cathartic and therapeutic potential to this approach, aligned with similar findings in the work psychology field [16]. Also, our finding supports the idea that audio diaries could reveal “hidden private phenomenon” [48] in sleep and in circadian rhythm research given participants’ ability to tailor their use based on their circadian preferences. However, we need to acknowledge that (as with any other method encouraging participants’ reflection) the act of completing the diary could, and indeed seemed to, encourage changes to their routine. This could be seen as helpful for a wider clinical application in both PA and sleep interventions. Despite participants reporting some initial concerns in providing the “right” information and occasional discouragement from awareness of their behaviours, they appreciated the ease and practicality of the method. Instant recording and the short time needed to record each audio diary entry contributed to the acceptability and 97% completion rate of the study. While previous studies with paper diaries and PA monitors recorded lower rates of daily data entries [49, 50]. Audio diaries were also described as inclusive, overcoming barriers related to visual impairments or physical limitations. Thus, here we support the use of audio diaries in research to possibly overcome issues related to completion and motivation in conjunction with other research methods (such as interviews and/or quantitative measures). Overall, our findings suggest that audio diaries were a relatively inclusive, easy to use, and flexible approach to explore movement behaviours when qualitative accounts and perspectives of people with lived experience are needed to inform development of recommendations and interventions for health support and promotion.

The study has some limitations. First, the use of audio diaries for a 7-day period is not able to capture a participant’s behavioural pattern over a longer term. In particular, in people living with SMI, routines and condition status fluctuate substantially. While a 7-day approach is therefore perhaps not optimal to capture data that could be used to infer typical levels of movement and sleep behaviours, the ease of audio diaries may lead them to working well for even more extended periods of data capture in future work. Another factor to consider is that our findings are not generalisable or representative of all the experiences people living with SMI might have in relation to sleep, PA and sedentary time. For example, we have not been able to recruit people with learning disabilities and from different ethnicities. Future research should take into account different cultural meanings of mental health impact. Lastly, the presence of a self-selection bias should be accounted for [51]. Future studies involving volunteers should consider personal preferences and attitudes of people living with SMI towards PA, and sedentary time.

## Conclusion

This study provides a unique insight into the usability and acceptability of audio diaries to explore dynamics and experiences of PA, sedentary time, and sleep within a 24 h framework among individuals living with SMI. Our findings showed how a participatory and flexible approach to data collection accounting for participants’ perceptions and life contexts can reveal the complex interplay between mental and physical health, and how these impacted movement behaviours in relation to both movement opportunities and sleep quality. Commonly reported challenges to movement and good sleep were medication side effects and mental or physical illness symptoms. While participants shared experiences of internal struggles and guilt feelings regarding their movement routines, walking, being outdoor and purposeful activities emerged as factors which contribute to enhanced mental wellbeing and sense of fulfilment. Here, we argue that a shift towards emphasizing the purpose and enjoyment derived from completing a movement activity rather than targets, might promote and support PA engagement in this group. Similarly, a more holistic approach considering the impact of disrupted and poor-quality sleep on movement opportunities is needed. Co-production with inputs from people with lived experience in sleep hygiene and PA recommendations, along with tailored sleep promotion for individuals living with SMI, is fundamental to better target public health messaging. The use of audio diaries offered an easy to use, acceptable, and relatively inclusive method to explore 24 h movement behaviours in research with people living with SMI.

## Electronic supplementary material

Below is the link to the electronic supplementary material.

Supplementary Material 1

Supplementary Material 2

Supplementary Material 3

## Data Availability

No datasets were generated or analysed during the current study.

## Abbreviations

SMI: Severe mental illness
PA: Physical activity
