# Supplementary material for: Speak for yourself: usability and acceptability of audio diaries to explore physical activity, sedentary and sleep behaviours of those living with severe mental illness
Source: J Act Sedentary Sleep Behav. 2024 Sep 11;3:21. doi: 10.1186/s44167-024-00058-4 (PMC11960249; doi:10.1186/s44167-024-00058-4)
Supplement: Supplementary file 1 — Supplementary Material 1 [file 44167_2024_58_MOESM1_ESM.pdf]

Participants' self-written free-text qualitative anonymised descriptions

Participant 1: *I am a female in my thirties with English background. I'm not religious, and have 2 children; I have lived experience of retail and familial experience of a range of mental health conditions including Complex PTSD, anxiety and depression, my son and partner also have ADHD so I understand a lot about that.*

Participant 2: *I am a transgender woman to male person, in my thirties, I was born in the United Kingdom and have lived in North east all of my life. I don't have any religions. I have lived experience in mental health in all different areas.*

Participant 3: *I am a 33 year old cis male. Who has lived experience of severe mental illness as well as using this experience to work in mental health as a peer supporter and therapeutic enabler.*

Participant 4: I *am a female in my 50's with a background in nursing, social work and counselling. I have also lived experience of mental health services for past 40 years. I'm autistic and have ADHD. I also suffer from chronic physical health problems.*

Participant 5: *I am a white British cis female. I am recently widowed. I have no religious beliefs. I have independent adult children. Regarding mental health issues within my family these are relatively minor - anxiety and depression. Our family is a big believer in the benefits of therapy, self care, communication and reflection.*

Participant 6: *I am a cis female, with a white British background. I was born in the North East of England and have lived here all of my life. I am a single parent to two daughters, and do not class myself as religious. I have lived experience of depression, anxiety, postnatal depression and living with a personality disorder.*

Participant 7: *I am a female in her 60s. Unfortunately I was assaulted and injured whilst working and have suffered with physical and mental illness since the day of the assault. I struggle with daily life and motivation. I am a caring person and enjoyed caring and supporting people. I have a grown up son and am married, my husband is now my carer.*

Participant 8: *I'm a female who is in her 50s. I am catholic religion and have lived experience of mental illness and also have family members with mental illness.*

Participant 9: *I am a white British female. I am married and have 2 children. I am not religious. My working background is in social care. I have lived experience of autism and psychosis.*
