# Supplementary material for: Speak for yourself: usability and acceptability of audio diaries to explore physical activity, sedentary and sleep behaviours of those living with severe mental illness
Source: J Act Sedentary Sleep Behav. 2024 Sep 11;3:21. doi: 10.1186/s44167-024-00058-4 (PMC11960249; doi:10.1186/s44167-024-00058-4)
Supplement: Supplementary file 2 — Supplementary Material 2 [file 44167_2024_58_MOESM2_ESM.pdf]

Audio diary guide

PASS24 SMI - Understanding what matters for people living with severe mental illness – a 24h approach to physical activity, sedentary behaviour and sleep

Lead Researcher: Dr Ilaria Pina, Newcastle University

Contact details: ilaria.pina@newcastle.ac.uk

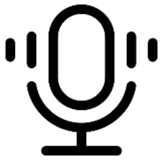

Thanks for agreeing to record an audio diary as part of the PASS24 SMI project.

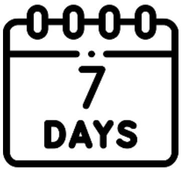

This will involve recording a brief summary of your physical activity, sedentary time, and sleep for a period of 7 days.

You can record the diary entry either during the day or at the end of each day, it is completely up to you and your preferences.

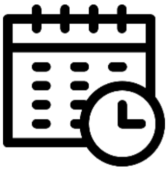

The following notes provide a guide to the sort of information you may include, but feel free to record any points about your experiences of physical activity, sedentary time, and sleep or other factors which may influence your current behaviours.

For each diary entry, please record day, date, and time.

Please find below some prompts and suggestions for your diary entry:

- Tell me about your physical activity, sedentary time, sleep (including napping) today.
- What made you decide to do (or not to do) some physical activity today?
- Did you have any positive physical activity/movement experience today?
- Are you having any distress or fatigue in moving today?
- Did you have much relaxation today?
- Did anything unexpected come up today in relation to physical activity or sleep?
- Did you nap today? What made you decide or not to nap?

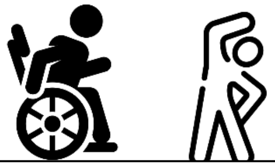

Physical activity: all movement including during leisure time, for transport to get to and from places, or as part of a person's work. Exercise is a subset physical activity that is planned and structured.

Sedentary behaviour: any waking behaviour with low energy expenditure, while in a sitting, reclining, or lying posture (or moving in a power chair or being pushed in a wheelchair).

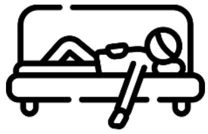

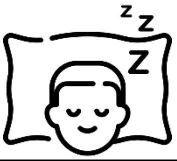

Sleep: resting state in which the body is not active, and the mind is unconscious.
Nap: short sleep period, usually taken during the day, apart from a person's primary sleep period.

We understand that talking about physical and mental health issues can cause distress for some people. If you think you have been affected by any of the topics discussed as part of the research, support is available to you. We would guide you to talk to one of the services listed below. You can also contact a member of the research team, using the email address below.

- Samaritans - 0330 094 5717
- Mind Infoline – 0300 123 3393
- Cumbria Crisis Resolution Team– 0800 652 2865
- Newcastle and Gateshead Crisis Resolution Team - 0800 652 2863
- Sunderland and South Tyneside Crisis Resolution Team - 0800 652 2867
- Northumberland and North Tyneside Crisis Resolution Team - 0800 652 2861

Researcher: Dr Ilaria Pina, Ilaria.pina@newcastle.ac.uk
