# Supplementary material for: Speak for yourself: usability and acceptability of audio diaries to explore physical activity, sedentary and sleep behaviours of those living with severe mental illness
Source: J Act Sedentary Sleep Behav. 2024 Sep 11;3:21. doi: 10.1186/s44167-024-00058-4 (PMC11960249; doi:10.1186/s44167-024-00058-4)
Supplement: Supplementary file 3 — Supplementary Material 3 [file 44167_2024_58_MOESM3_ESM.pdf]

Entrance interview guide

PASS24 SMI - Understanding what matters for people living with severe mental illness – a 24h approach to physical activity, sedentary behaviour and sleep

ICE BREAKER: Please could you introduce yourself, mention a bit about your background, and give a general overview of your interest in physical activity and sleep?

Physical activity

What does be able to be physically active mean to you?

Could you please describe your understanding about the impact of physical activity on your health?

Did you receive any physical activity support/advice from health professionals in the past? If yes, could you please provide an example? (If no, would you like to receive more support from health professionals on this aspect?)

What challenges have you encountered or are currently facing to be physically active?

Sedentary time

Could you please describe the most common factors that affect your energy levels?

Sleep

Could you please describe your understanding about the impact of sleep on your health?

Did you receive any sleep support/advice from health professionals in the past? If yes, could you please provide an example? (If no, would you like to receive more support from health professionals on this aspect?)

What challenges have you encountered or are currently facing to have good quality sleep?

CLOSING: Is there anything else you would like to mention that hasn't come up in the questions we've asked?

Exit interview guide

PASS24 SMI - Understanding what matters for people living with severe mental illness – a 24h approach to physical activity, sedentary behaviour and sleep

---

Thank you for taking part in this study and submitting your diary entries.

Can you tell me about your experience of completing an audio diary?

Which part of the research did you enjoy participating in the most – Audio diaries or earlier interview? Please give reasons for your choice.

Did you encounter any difficulties when participating in the audio diary?

Do you think audio diaries or written diaries are more suitable in capturing your experiences?’

CLOSING: Is there anything else you would like to mention that hasn’t come up in the questions we’ve asked?
